# Follower proactive behavior reducing supervisory abuse: the role of leader future orientation and follower in-role performance

**DOI:** 10.3389/fpsyg.2026.1916692

**Published:** 2026-09-01

**Authors:** Qin Xu, Shuming Zhao, Fangjun Li, Jiapeng Wang, Yuxin Zou, Iqbal Fajri Albani

**Affiliations:** 1 School of Economics and Management, Southeast University, Nanjing, China; 2 School of Business, Nanjing University, Nanjing, China; 3 School of Management, Jinan University, Guangzhou, China

**Keywords:** abusive supervision, future orientation, in-role performance, leader future orientation, proactive behavior

## Abstract

**Introduction:**

Despite growing interest in leaders’ responses to employee proactivity, it remains largely unanswered whether and when subordinate proactive behavior mitigates supervisory abuse.

**Methods:**

We propose a model grounded in the logic of moral exclusion, in which subordinate proactive behavior reduces supervisory abuse, magnified by the supervisor’s future orientation and the subordinate’s in-role performance. We test the proposed model through a multi-source field study (Study 1, involving 191 employees and 86 supervisors in an import and export company) and a 2 × 2 vignette experiment (Study 2, with 243 employee participants from various industries).

**Results:**

Our findings reveal a main effect in which subordinate proactive behavior indeed reduces supervisory abuse. Additionally, we observe a three-way moderating effect, in which the enhancement of a supervisor’s future orientation on the negative association between subordinate proactive behavior and abusive supervision becomes more pronounced for subordinates with high in-role performance.

**Discussion:**

These findings further our existing understanding of coping strategies for supervisory abuse and managerial responses to employee proactive behavior.

## Introduction

Extant leadership research has predominantly adopted a top-down perspective, emphasizing how leaders shape subordinates’ attitudes and behaviors. An emerging stream of research has reversed this lens by examining how followers’ values, characteristics, and behaviors influence leaders’ cognitions and actions, an approach referred to as followership (Bhattacharjee and Sarkar, 2024; Smith et al., 2026). Within this literature, an increasingly important question concerns how a leader interprets, evaluates, and responds to employee proactive behavior (Gajendran et al., 2024; Huang et al., 2023). Employee proactive behavior refers to self-initiated, future-focused, and change-oriented actions through which the employee seeks to influence themselves or their work environments (Parker and Collins, 2010). It encompasses a broad range of discretionary activities, including anticipating and preventing potential problems, generating and implementing useful ideas, improving work procedures, and initiating constructive change (Li et al., 2025; Liu et al., 2024).

Despite growing interest in leaders’ responses to employees’ proactivity, an important question remains largely unanswered: Can employee proactive behavior reduce the likelihood of abusive supervision? Abusive supervision, defined as supervisors’ sustained display of hostile verbal and nonverbal behaviors excluding physical contact, has detrimental consequences for both employees and organizations (Fischer et al., 2021; Tepper, 2000). Yet research on the bottom-up consequences of employee proactive behavior has focused primarily on leaders’ evaluations of, attitudes toward, and supportive responses to proactive employees, including performance evaluations, liking, trust, endorsement, and empowering leadership (e.g., Grant et al., 2009; Han et al., 2019). Consequently, we know considerably less about whether constructive employee behavior can shape and potentially constrain the emergence of destructive leadership behavior.

We address this question through moral exclusion theory (Opotow, 1990, 1995). The theory proposes that people apply moral rules and standards of fairness within a psychologically defined scope of justice. Targets perceived as useful for attaining important goals are more likely to remain within this moral boundary and to be treated with dignity and restraint; targets perceived as obstructive or expendable are more vulnerable to moral exclusion and mistreatment. This utility-based logic is particularly relevant to supervisor-subordinate relationships because supervisors depend on employees to accomplish unit goals. Employee proactive behavior can increase the employee’s utility by helping the supervisor detect problems, respond to change, and improve future functioning (Carsten et al., 2018; Parker and Collins, 2010). We therefore propose that proactive employees are more likely to be included within supervisors’ scope of justice and, consequently, less likely to experience abusive supervision.

**Figure 1:**
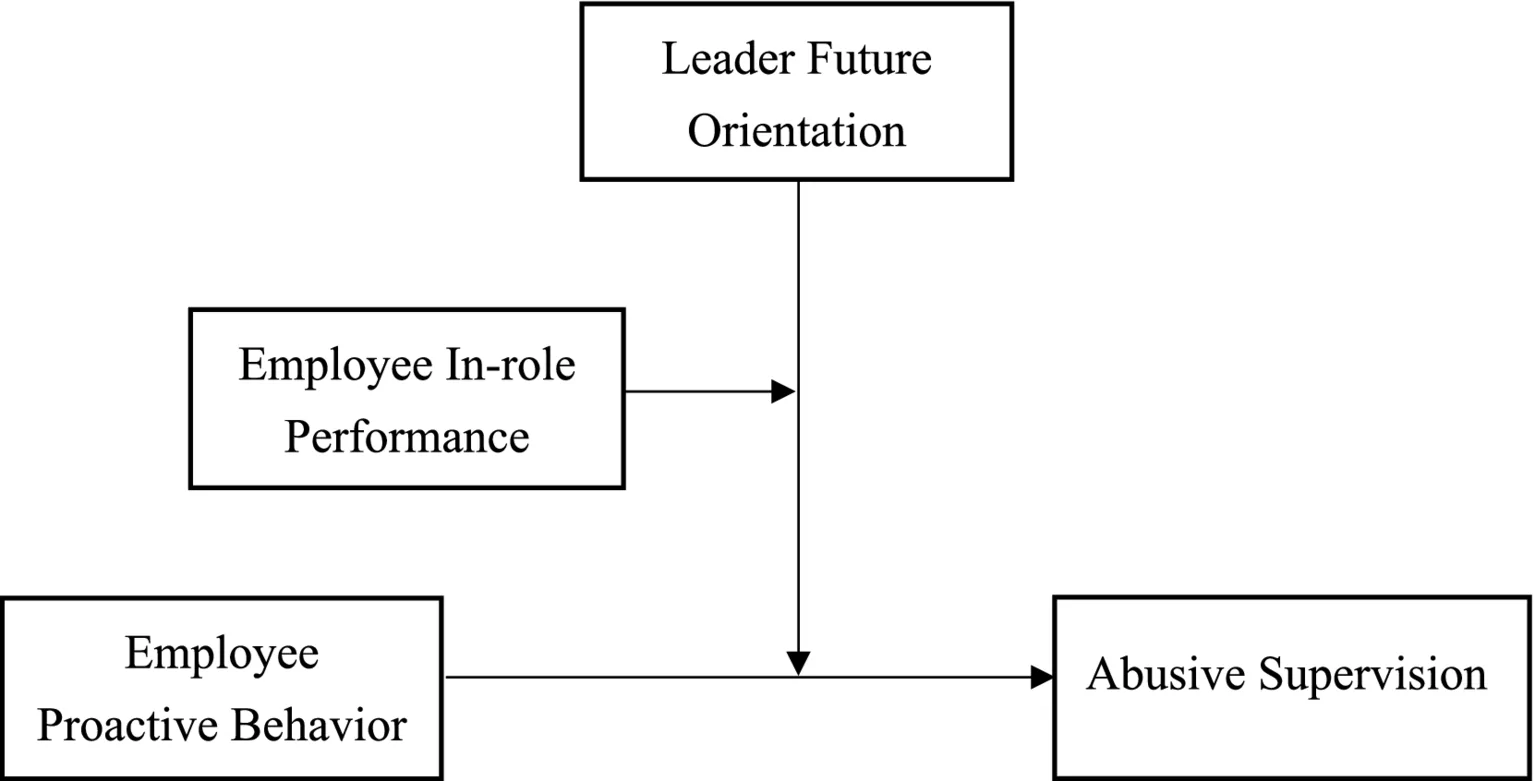
Conceptual model.

The utility conveyed by proactivity, however, is neither universal nor self-evident. First, it depends on what the supervisor values. Because proactive behavior is inherently oriented toward future problems and possibilities, it should be especially useful to supervisors who strongly consider the future consequences of immediate actions. We therefore predict that supervisor future orientation strengthens the negative relationship between employee proactive behavior and abusive supervision. Second, the utility of proactive actions depends on whether the employee appears capable of converting initiative into effective outcomes. Employee in-role performance can provide supervisors with concrete evidence of competence and reliability. Thus, the protective association of proactivity is strongest when a future-oriented supervisor evaluates the employee who also performs core duties well. Taken together, we propose a three-way interaction model in which the effect of employee proactivity on abusive supervision is jointly moderated by employee in-role performance and supervisor future orientation, as shown in Figure 1.

The contributions of the present study are threefold. Our first contribution lies in extending the followership studies in the area of proactive behavior by pinpointing abusive supervision as an under-explored leader response. Research on leader responses to subordinate proactivity has been mainly limited to various supervisor evaluations of subordinates and attitudes towards them, such as performance evaluation, learning rating, help behavior rating, and leader liking (e.g., Duan et al., 2021; Han et al., 2019; Lin et al., 2024). However, only a limited number of studies have explicitly examined leadership itself, such as leader motivation (Carsten et al., 2018) and empowerment (Han et al., 2019). The current study attempts to enrich this latter research stream by pinpointing supervisory abuse as a possible outcome (*what* referred by Whetten, 1989).

Second, we clarify when and why employee proactive behavior may constrain abusive supervision. Although leaders are not always receptive to proactive employees (Parker et al., 2019), prior research suggests that their reactions depend on contextual conditions. Proactivity may elicit unfavorable evaluations when leaders lack felt responsibility for change or proactive personality, but may generate endorsement and favorable performance evaluations when it is perceived as constructive and valuable (e.g., Grant et al., 2009). Building on the general tendency for leaders to value proactive employee’s characteristics (Benson et al., 2016), we propose that employee proactive behavior is negatively related to abusive supervision. We further identify supervisor future orientation and employee in-role performance as joint boundary conditions, thereby advancing the understanding of social and relational context underlying wise proactivity (Parker et al., 2019).

The third contribution is suggesting employee proactive behavior as a promising coping strategy for supervisory abuse, responding to the call for investigations of subordinates as active players who reduce (or even stop) abusive supervision from the perspective of followership (a systematic review by Bhattacharjee and Sarkar, 2024, p. 24). Subordinate behaviors are considered a major driver of supervisory abuse (Fischer et al., 2021). Negative subordinate behaviors, such as deviance, counterproductive actions, and aggression, have been identified as significant triggers for abusive supervision (e.g., Lyubykh et al., 2022; Walter et al., 2015). However, there is a paucity of studies examining how employees can actively and effectively cope with supervisory abuse (Wee et al., 2017). By exploring a shielding effect of proactive behavior against supervisory abuse, we attempt to advance this line of research.

## Literature review and hypotheses development

### Moral exclusion theory

Moral exclusion theory explains why moral values, rules, and considerations of fairness are not applied equally to all targets (Opotow, 1990, 1995). The theory proposes that individuals construct a psychological boundary-the scope of justice or moral community-within which others are regarded as deserving of fair, respectful, and humane treatment. Targets located outside this boundary receive less moral consideration; as a result, neglect, humiliation, exploitation, or aggression toward them may become easier to rationalize. Moral exclusion does not necessarily require an explicit judgment that a target is immoral or less human. It can also take subtler forms in which the target’s welfare, rights, or dignity simply carry less weight in the perceiver’s decisions.

A central basis for moral inclusion is perceived utility. People are more likely to include targets who facilitate valued goals, provide important resources, or otherwise contribute to their interests, whereas targets who obstruct goal attainment or consume resources without sufficient contribution are more vulnerable to exclusion (Opotow, 1995; Tepper et al., 2011; Walter et al., 2015). Importantly, utility is relational rather than inherent. The same target behavior may be valued differently depending on the perceiver’s goals, time horizon, and judgment of the target’s capacity to produce useful outcomes. Moral inclusion therefore reflects not only what a target does, but also whether the perceiver values that contribution and believes the target can deliver it.

This logic has direct relevance to the emergence of abusive supervision. Supervisors depend on subordinates for the accomplishment of individual, team, and organizational goals. Prior research drawing on moral exclusion theory shows that supervisors’ dependence on subordinate performance can shape whether poorly performing subordinates are subjected to abuse (Tepper et al., 2011; Walter et al., 2015). Extending this reasoning, we argue that employee proactive behavior represents another consequential source of perceived utility. By anticipating problems and initiating improvements, employees can make themselves more instrumental to supervisors’ goals. This enhanced utility should increase moral inclusion and constrain abusive treatment. Moreover, because the value of proactivity depends on the supervisor’s goals and the employee’s demonstrated competence, moral exclusion theory provides a coherent basis for our proposed moderating roles of supervisor future orientation and employee in-role performance.

### Subordinate proactivity and supervisory abuse

Applying moral exclusion theory to supervisor-subordinate interactions, we propose that employee proactive behavior reduces abusive supervision. In increasingly dynamic work environments characterized by technological change, environmental uncertainty, and task complexity, supervisors often depend on proactive employees to identify emerging problems, improve existing practices, and facilitate the achievement of team and organizational goals (Qi et al., 2019; Zhang et al., 2025). Parker and Collins (2010) conceptualized proactive work behavior as a higher-order construct encompassing taking charge, voice, individual innovation, and problem prevention. Although these behaviors differ in their specific forms, they all involve employees taking self-initiated actions to improve internal organizational functioning and change the status quo.

Admittedly, employee proactivity does not invariably elicit favorable managerial responses. Some studies suggest that managers may react negatively to particular forms of proactivity that are highly challenging or threatening, or that are attributed to self-serving motives, such as prohibitive voice, status-challenging behavior, and initiatives perceived as impression management (Burris, 2012; Chamberlin et al., 2017; Duan et al., 2021). The present study, however, focuses on proactive behavior as a broad pattern of self-initiated, future-focused, and change-oriented actions, including anticipating and solving problems, generating and implementing useful ideas, and initiating constructive improvements (Parker and Collins, 2010). On average, such behavior is likely to help supervisors fulfill their responsibilities and attain important work goals. It may also signal that an employee possesses initiative, responsibility, competence, and commitment, thereby enhancing the employee’s perceived utility to the supervisor.

Existing evidence supports the instrumental value of employee proactivity. Voluntary proactive contributions can facilitate knowledge sharing (Liu et al., 2024), promote group innovation, and enhance organizational service quality (Li et al., 2025). Moreover, implicit followership research indicates that leaders generally regard “going above and beyond” and being a good organizational citizen as defining characteristics of an effective follower (Sy, 2010). Thus, proactive employees are likely to be perceived not merely as performing discretionary behaviors, but as helping supervisors address work demands and accomplish valued objectives.

According to moral exclusion theory, such utility judgments shape whether a target is included within the perceiver’s scope of justice. Employees who contribute to supervisors’ goal attainment are more likely to be viewed as deserving of fairness, dignity, and moral consideration. Consequently, highly proactive employees should be more readily included within supervisors’ moral communities, making hostile or demeaning treatment less psychologically acceptable. In contrast, employees who rarely anticipate problems, initiate improvements, or respond constructively to changing demands may be perceived as less useful, or even as impediments to supervisors’ goal attainment. They may therefore be more vulnerable to moral exclusion, which can lower supervisors’ moral restraints against hostile verbal and nonverbal behaviors, such as belittling, humiliating, or ignoring subordinates (Emmerling et al., 2023). Therefore, we hypothesize that,

*Hypothesis 1: Employee proactive behavior is negatively related to supervisory abuse*.

### Moderating role of supervisor future orientation

Moral exclusion research suggests that a target’s perceived utility depends on the perceiver’s goals and on the extent to which the perceiver relies on the target for goal attainment (Stephan et al., 2024; Walter et al., 2015). Accordingly, supervisors may differ in the value they assign to employee proactive behavior. We propose that supervisor future orientation strengthens the negative relationship between employee proactive behavior and abusive supervision.

Future orientation refers to an individual’s tendency to consider the future consequences of present actions and to attach importance to future goals and outcomes (Strathman et al., 1994). Highly future-oriented supervisors are particularly concerned with narrowing discrepancies between current conditions and desired future states (Choi et al., 2023). They are also more receptive to work environments and employee actions that emphasize changing the status quo (Strauss and Parker, 2018) and place greater emphasis on future expectations and the attainment of long-term goals (Zhang et al., 2026). These priorities closely correspond to the anticipatory, future-focused, and change-oriented nature of employee proactive behavior.

For highly future-oriented supervisors, proactive employees therefore represent a particularly relevant and valuable resource. By identifying emerging problems, anticipating future demands, and initiating constructive improvements, proactive employees help supervisors address potential threats and move their units toward desired future states. Highly future-oriented supervisors should consequently be more likely to recognize the potential utility conveyed by proactive behavior (Kristof-Brown and Guay, 2011). Employee proactivity should thus create a pronounced difference in perceived utility between employees who actively facilitate future goal attainment and those who remain passive until problems become immediate. From a moral exclusion perspective, this difference in perceived utility should translate into a stronger distinction in moral inclusion. Highly proactive employees are more likely to be viewed as deserving of fair and respectful treatment, whereas less proactive employees may be perceived as failing to support, or even impeding the supervisor’s future-oriented goals. The negative relationship between employee proactive behavior and abusive supervision should therefore be particularly strong among highly future-oriented supervisors.

By contrast, supervisors with lower future orientation focus more heavily on current operations and immediate task completion. Although they may still appreciate useful employee initiative, anticipatory and change-oriented contributions are less central to their goals. Employee proactive behavior should therefore carry less weight in their judgments of employee utility and moral deservingness. Moreover, because proactive behavior often involves challenging existing practices and initiating change, supervisors who attach limited value to future-oriented improvement may be more likely to perceive such behavior as disruptive or threatening, potentially generating interpersonal tension (Tepper et al., 2011). Nevertheless, our argument does not require the relationship between employee proactivity and abusive supervision to reverse. Rather, the difference in abusive treatment between highly proactive and less proactive employees should be smaller because supervisors with lower future orientation have weaker goal-based reasons to regard proactivity as an especially valuable contribution. Therefore, we propose:

*Hypothesis 2: Leader future orientation moderates the relationship between employee proactive behavior and abusive supervision, such that the relationship is stronger when leader future orientation is high*.

### Joint roles of employee in-role performance and leader future orientation

Although supervisor future orientation determines the extent to which future-focused employee contributions are valued, employee in-role performance helps determine whether those contributions are perceived as credible and realizable. Employees’ abilities and competencies constitute an important basis for supervisors’ assessments of the utility of employee proactivity (Bhattacharjee and Sarkar, 2024; Walter et al., 2015). In-role performance reflects the extent to which employees effectively fulfill their formally prescribed responsibilities and produce expected work outcomes. Because successful task performance provides visible evidence of competence, reliability, and execution capability, it should shape how supervisors interpret the value conveyed by proactive behavior. We therefore propose that the moderating effect of supervisor future orientation is more pronounced when employee in-role performance is high.

When employees consistently meet or exceed in-role performance expectations, their proactive behavior is more likely to be interpreted as a credible extension of demonstrated competence. High-performing employees have already shown that they can effectively manage their current responsibilities. Their proactive initiatives are therefore less likely to be dismissed as poorly informed interference, unrealistic suggestions, or distractions from core duties. Instead, such initiatives indicate that employees can both perform their prescribed tasks and contribute beyond formal requirements. For highly future-oriented supervisors, this combination is particularly valuable because it signals that employees are capable of delivering present results while also helping create desired future consequences. Proactivity is therefore both aligned with the supervisor’s future-oriented goals and supported by evidence that the employee can translate initiative into effective action (Wee et al., 2017). Consistent with evidence that employee performance strengthens the trust-related benefits associated with proactivity (Han et al., 2019), this configuration should maximize the employee’s perceived utility, facilitate inclusion within the supervisor’s scope of justice, and reduce the likelihood of abusive treatment.

The pattern should differ among supervisors with lower future orientation. Because these supervisors place greater emphasis on current operations and immediate outcomes than on long-term improvement (Choi et al., 2023), strong in-role performance may be sufficient to establish an employee’s utility. Even when a high-performing employee behaves proactively, the employee’s anticipatory and change-oriented contributions are less closely aligned with the supervisor’s priorities and therefore provide less additional value. Consequently, the negative relationship between employee proactive behavior and abusive supervision should be weaker when supervisor future orientation is low, even among high-performing employees.

When employee in-role performance is low, proactive behavior conveys a more ambiguous signal. The supervisor may question whether the employee possesses the knowledge, discipline, or execution capability required to implement proposed changes successfully. They may also perceive proactive initiatives as diverting attention from core responsibilities or creating additional demands that the employee is not equipped to manage. Under these conditions, even highly future-oriented supervisors have weaker grounds for interpreting employee proactivity as a credible contribution to future goal attainment. The lack of cognitive trust in low-performing employees’ abilities should therefore reduce the extent to which supervisor future orientation differentiates responses to their proactive behaviors (Han et al., 2019). As a result, the interaction between employee proactive behavior and supervisor future orientation should be weaker when employee in-role performance is low.

These arguments suggest a three-way interaction among employee proactive behavior, supervisor future orientation, and employee in-role performance. The negative relationship between employee proactive behavior and abusive supervision should be strongest when supervisor future orientation and employee in-role performance are both high. Under this condition, proactivity is closely aligned with the supervisor’s future goals and validated by the employee’s demonstrated competence. When supervisor future orientation is low, proactivity is less relevant to the supervisor’s priorities; when employee in-role performance is low, proactivity is less credible as a realizable contribution. Either condition should weaken the extent to which employee proactive behavior reduces abusive supervision. Based on this line of reasoning, we propose:

*Hypothesis 3: Employee in-role performance moderates the positive interactive effect of employee proactive behavior and leader future orientation on abusive supervision in such a way that this interaction only occurs for employees with high in-role performance*.

## Overview of current research

According to Wright and Sweeney’s (2016) suggestions, we implemented a multi-study design to test our hypothesized model. Specifically, we employed a combination of a multi-source field survey (Study 1) and a vignette experiment (Study 2). The multi-source field survey has two primary advantages: first, it reduces common method bias, and second, it offers a degree of ecological validity (Podsakoff et al., 2003). However, due to the cross-sectional nature of the data, it does not allow for robust causal inferences. Additionally, although Study 1 is a field study, it relies on data from a single organization, which restricts the generalizability of the findings. The vignette experiment helps address these limitations by manipulating independent variables (employee proactivity × in-role performance) and observing subsequent changes in the dependent variable (supervisory abuse), thus enhancing causal inference (Fischer et al., 2021) using a sample drawn from various organizations. Previous studies have used similar procedures (e.g., Camps et al., 2020, Study 2b, p. 505; Walter et al., 2015, Study 1, p. 1060). By using these two complementary research methods, we aim to strengthen the replicability and generalizability of our findings (Podsakoff et al., 2012).

### Study 1 method

#### Sample and procedure

Employees and their direct supervisors from a large company in Southeast China were invited to participate in Study 1. Separate survey packages were distributed to supervisors and their subordinates. Supervisors rated their subordinates’ proactive behavior and in-role performance, their own future orientation, as well as demographic variables. Subordinates evaluated their supervisors’ abusive supervision and their demographic variables. Completed surveys were returned directly to the researchers using pre-stamped envelopes contained in the packages.

We distributed questionnaires to 250 employees and their 93 direct supervisors. After deleting incomplete and unmatched responses, 191 employees and 86 supervisors remained (each supervisor evaluates an average of 2.2 subordinates), yielding a valid response rate of 76.4% for employees and 92.5% for supervisors. 26.7% of the participating employees were male (SD = 0.44), with an average organizational tenure of 4.42 years (SD = 3.86). 11% had a vocational degree or lower, 74.9% had a bachelor’s degree and 14.1% had a master’s degree or higher (SD = 0.53); 63.4% were at or below the age of 30 (SD = 0.98). Of the supervisors, 47.1% were men (SD = 0.50), and average organizational tenure was 13.98 years (SD = 7.67). 17.2% had a vocational degree or lower, 57.5% had a bachelor’s degree 25.3% had a master’s degree or higher (SD = 0.71); 58.6% aged from 31 to 40 years, and 26.4% were aged from 41 to 50 years (SD = 1.35). The duration of supervisor-subordinate working relationships is as follows: 33 pairs (18.3%) have worked together for less than 1 year, 82 pairs (42.9%) for 1–3 years, 33 pairs (17.3%) for 3–5 years, and 41 pairs (21.5%) for more than 5 years.

#### Measures

Following the back-translation procedure, all the English items were translated into Chinese. A Likert-type scale ranging from 1 (*strongly disagree*) to 7 (*strongly agree*) was used for all the measures.

##### Employee proactive behavior

We used a 10-item scale from Parker and Collins (2010) to measure proactivity. Sample items included “This subordinate tries to find the root cause of things that go wrong,” and “This subordinate speaks up and encourages others in the workplace to get involved with issues that affect us.” Cronbach’s alpha was 0.96.

##### Supervisor future orientation

A five-item scale developed by Strathman et al. (1994) was used to measure future orientation. A sample item was “I consider how things might be in the future and try to influence those things with my day-to-day behavior.” Cronbach’s alpha was 0.75.

##### Employee in-role performance

A four-item scale developed by Williams and Anderson (1991) was used to measure employee performance. A sample item was “This subordinate adequately completed assigned duties.” Cronbach’s alpha was 0.96.

##### Abusive supervision

An abridged version (a five-item scale, Mitchell and Ambrose, 2007) adapted from Tepper (2000) was used to measure active abusive supervision. A sample item included “My supervisor makes negative comments about me to others.” Cronbach’s alpha was 0.91.

##### Control variables

We controlled for employee gender, organizational tenure, supervisor gender, organizational tenure, as well as team size because research suggested that these factors could influence the hostile work behaviors of leaders (Ouyang et al., 2015). Gender was dummy coded as 0 = male and 1 = female. Organizational tenure was measured with one item asking for the number of years in the organization.

#### Data analysis

Given that more than one subordinate reported to the same supervisor, HLM 6.08 was used to test our hypotheses (Raudenbush and Bryk, 2002). In this study, abusive supervision, employee proactive behavior, and in-role performance were Level 1 (subordinate-level) variables, and leader future orientation was Level 2 (supervisor-level) variables. According to Hofmann and Gavin’s (1998) suggestions, our Level 1 predictors were group-mean centered because they helped separate out the cross-level and between-group interactions. The Level 2 variable was grand-mean centered because it allowed us to reduce the potential problems associated with multicollinearity.

### Study 1 results

#### Confirmatory factor analysis

Confirmatory factor analyses (CFAs) were conducted to examine whether it was appropriate to treat three subordinate-related variables as distinct constructs. We compared the three-factor model with four alternative nested models. As shown in Table 1, the three-factor model (subordinate proactive behavior and in-role performance, subordinate-rated abusive supervision) demonstrated an acceptable fit (*χ*^2^(132) = 407.24, CFI = 0.93, SRMR = 0.046). Moreover, this model achieved a significantly better fit than all the alternative models. Therefore, the three constructs exhibited satisfactory discriminant validity.

**Table 1:** Results of confirmatory factor analysis (Study 1).

Model	*χ* ^2^	*df*	CFI	SRMR	Δ*χ*^2^
Three-factor model	**407.24**	**132**	**0.93**	**0.046**	
Two-factor model a	1084.88	134	0.80	0.066	*χ*^2^ (2) = 677.64^***^
Two-factor model b	1389.86	134	0.72	0.18	*χ*^2^ (2) = 982.62^***^
Two-factor model c	1402.94	134	0.72	0.18	*χ*^2^ (2) = 995.70^***^
Single-factor model	2044.40	135	0.59	0.18	*χ*^2^ (3) = 1637.16^***^

Values in bold indicate the baseline model; two-factor model a: proactive behavior and in-role performance combined; two-factor model b: proactive behavior and abusive supervision combined; two-factor model c: in-role performance and abusive supervision combined. ***p < 0.001.

#### Descriptive statistics and reliability test

Descriptive statistics and correlations for all study variables in Study 1 are presented in Table 2. As shown, employee gender was significantly correlated with proactive behavior. Therefore, we tested the hypotheses both with and without control variables. We presented results with control variables included to provide full information. Moreover, employee proactive behavior was negatively related to abusive supervision (*r* = −0.24, *p* < 0.01). This finding is consistent with Hypothesis 1. Besides, average variance extracted (AVE) and composite reliability (CR) are presented in Table 3. All AVE values are not smaller than 0.5 and all CR values are >0.7. Thus, the measures of this study are reliable.

**Table 2:** Means, standard deviations, and correlations (Study 1).

Variables	Mean	SD	1	2	3	4
Individual level						
1. Employee gender	0.73	0.44	–			
2. Employee organizational tenure	4.42	3.86	−0.07	–		
3. Employee proactive behavior	4.99	1.02	0.19^**^	0.13	–	
4. Employee in-role performance	5.43	0.97	0.12	0.11	0.67^**^	–
5. Abusive supervision	2.14	1.06	−0.14	−0.13	−0.24^**^	−0.18^*^
Group level						
1. Supervisor gender	0.53	0.50	–			
2. Supervisor organizational tenure	13.98	7.67	−0.14	–		
3. Team size	2.21	1.38	−0.09	−0.08	–	
4. Supervisor future orientation	5.57	0.80	−0.04	0.09	0.03	–

Individual level *N* = 191, group level *N* = 86; *p < 0.05, **p < 0.01.

**Table 3:** Results of the reliability analysis (Study 1).

Variables	Items	Loading	Cronbach’s alpha	AVE	CR
Proactive behavior (Parker and Collins, 2010)	1. This subordinate generates creative ideas.	0.87	0.96	0.59	0.94
	2. This subordinate searches out new techniques, technologies and/or product ideas.	0.73			
	3. This subordinate promotes and champions ideas to others.	0.68			
	4. This subordinate communicates his/her views about work issues to others in the workplace, even if his/her views differ and others disagree with him/her.	0.83			
	5. This subordinate speak up and encourages others in the workplace to get involved with issues that affect him/her.	0.81			
	6. This subordinate keeps well informed about issues where his/her opinion might be useful to his/her workplace.	0.65			
	7. This subordinate speaks up with new ideas or changes in procedures.	0.77			
	8. This subordinate tries to bring about improved procedures in his/her workplace.	0.81			
	9. This subordinate tries to institute new work methods that are more effective.	0.77			
	10. This subordinate tries to implement solutions to pressing organization problems.	0.78			
In-role performance (Williams and Anderson, 1991)	1. This subordinate adequately completes assigned duties.	0.98	0.96	0.92	0.98
	2. This subordinate fulfills responsibilities specified in job description.	0.96			
	3. This subordinate performs tasks that are expected of him/her.	0.96			
	4. This subordinate meets formal performance requirements of the job.	0.95			
Abusive supervision (Mitchell and Ambrose, 2007)	1. My supervisor ridicules me.	0.83	0.91	0.75	0.94
	2. My supervisor tells me my thoughts or feelings are stupid	0.88			
	3. My supervisor puts me down in front of others	0.85			
	4. My supervisor makes negative comments about me to others	0.85			
	5. My supervisor tells me I’m incompetent	0.91			
Future orientation (Strathman et al., 1994)	1. I consider how things might be in the future and try to influence those things with my day to day behavior.	0.77	0.75	0.50	0.83
	2. Often, I engage in a particular behavior in order to achieve outcomes that may not result for many years.	0.73			
	3. I am willing to sacrifice my immediate happiness or well-being in order to achieve future outcomes.	0.72			
	4. I think it is important to take warnings about negative outcomes seriously even if the negative outcome will not occur for many years.	0.63			
	5. I think it is more important to perform a behavior with important distant consequences than a behavior with less-important immediate consequences.	0.67			

#### Tests of hypotheses

Table 4 presents the results. H1 stated that a subordinate’s proactive behavior was negatively related to abusive supervision. In Model 1, employee proactive behavior was significantly and negatively related to abusive supervision (*γ* = −0.26, *p* < 0.05). Hence, H1 was supported.

**Table 4:** HLM results for abusive supervision (Study 1).

Predictors	Model 1	Model 2	Model 3	Model 4
Control variables				
Emp gender	−0.23 (0.17)	−0.25 (0.17)	−0.22 (0.17)	−0.25 (0.17)
Emp Org	−0.03 (0.02)	−0.03 (0.02)	−0.03 (0.02)	−0.03 (0.02)
Sup Gender	0.14 (0.18)	0.14 (0.18)	0.19 (0.17)	0.18 (0.17)
Sup Org	−0.00 (0.01)	−0.00 (0.01)	−0.00 (0.01)	−0.00 (0.01)
Team size	0.04 (0.07)	0.04 (0.07)	0.06 (0.07)	0.05 (0.06)
Main effects				
PB	−0.26 (0.13)^*^	−0.26 (0.13)^*^	−0.30 (0.13)^*^	−0.31 (0.13)^*^
FO	0.15 (0.11)	0.14 (0.11)	0.15 (0.11)	0.25^*^ (0.12)
JP	0.04 (0.12)	0.03 (0.12)	0.02 (0.14)	0.05 (0.14)
Two-way interactions				
PB × FO		0.08 (0.12)	−0.18 (0.16)	−0.19 (0.16)
PB × JP			−0.08 (0.06)	−0.01 (0.07)
JP × FO			0.28 (0.16)	0.22 (0.16)
Three-way interaction				
PB × FO × JP				−0.17 (0.08)^*^

Robust standard errors are shown in parentheses; PB, subordinate proactive behavior; JP, employee in-role performance; FO, supervisor future orientation; Emp gender, employee gender; Emp Org, employee organizational tenure; Sup gender, supervisor gender; Sup Org, supervisor organizational tenure; Individual level *N* = 191; group level *N* = 87. ^*^p < 0.05; ^**^p < 0.01.

H2 proposed that a supervisor’s future orientation moderated the relationship between employee proactive behavior and abusive supervision. However, as shown in Model 2, the two-way interaction term of employee proactive behavior and supervisor future orientation was not significant (*γ* = 0.08, *p* = ns). H2 was, therefore, not supported.

H3 predicted a three-way interaction of employee proactive behavior, supervisor future orientation, and employee performance in predicting abusive supervision. In Model 4, the three-way interaction term was significantly related to abusive supervision (*γ* = −0.17, *p* < 0.05), after controlling the main effects and three two-way interactions.

**Figure 2:**
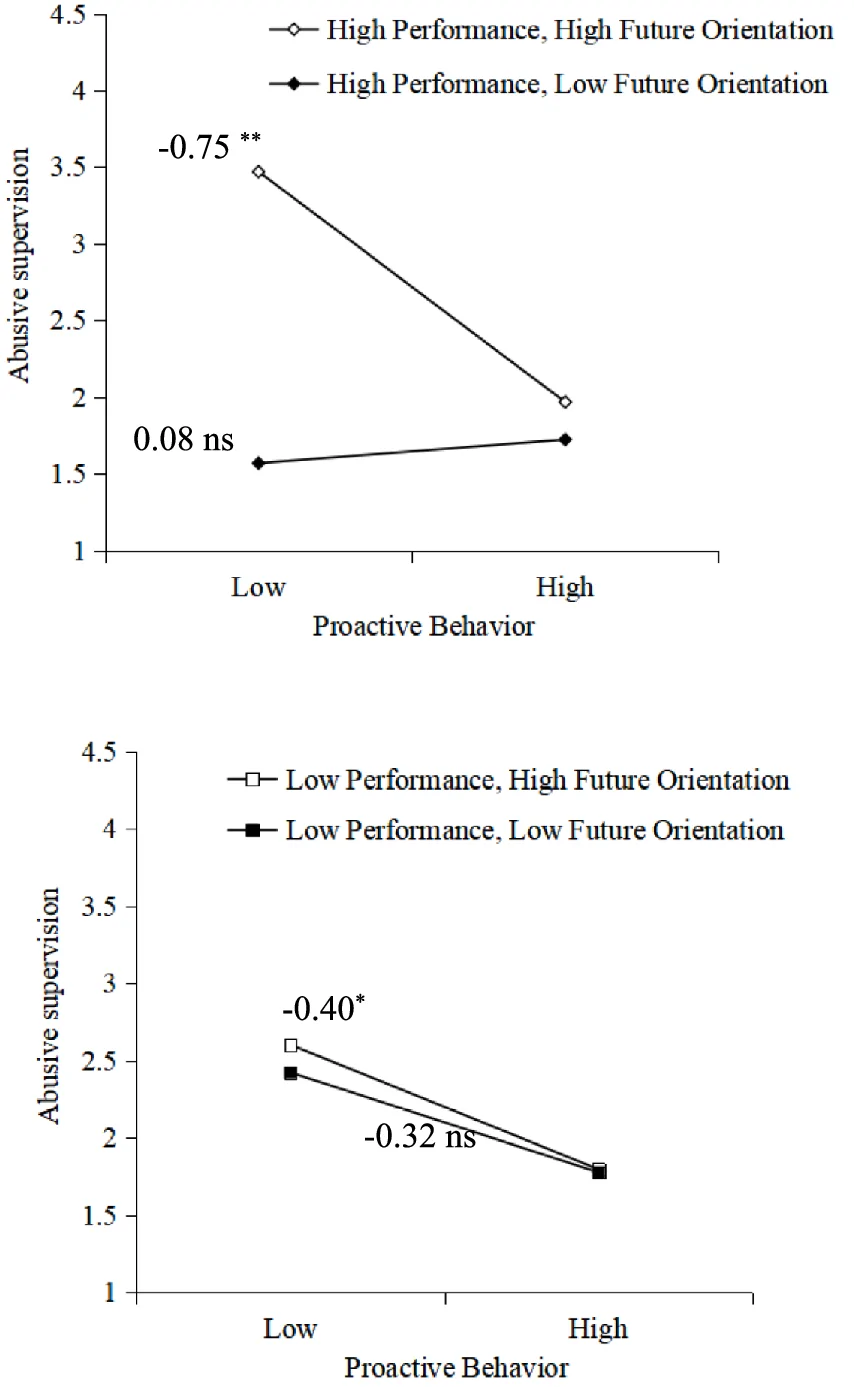
Three-way interaction effect on abusive supervision (Study 1).

Following the procedures proposed by Aiken and West (1991), we plotted the three-way interaction, as shown in Figure 2. Simple slope analyses indicated that the relationship between employee proactive behavior and abusive supervision was significantly negative when supervisor future orientation was high and employee in-role performance was high (*β* = −0.75, *p* < 0.01) but neutral when supervisor future orientation was low and employee in-role performance was high (*β* = 0.08, ns).

When employee in-role performance was low, employee proactive behavior was negatively associated with abusive supervision for those with lowly future-oriented supervisors (*β* = −0.40, *p* < 0.05) but was unrelated to abusive supervision for those with highly future-oriented supervisors (*β* = −0.32, p = ns). Slope difference analysis indicated that there was a significant slope difference in the employee proactive behavior- abusive supervision association when employee in-role performance and supervisor future orientation were both high versus when employee in-role performance was high and supervisor future orientation was low (*t* = −2.51, *p* < 0.05). On the contrary, the slope difference was not significant for employees with low in-role performance (*t* = −0.27, ns). H3 was thus supported.

While we did not discover a significant two-way interaction between employee proactive behavior and supervisor future orientation on abusive supervision, the results of our study lend support to the hypothesized three-way interaction pattern within this organizational context. Prior research has shown that abusive supervision can act as a precursor to employee proactive behavior by depleting employees’ psychological resources, diminishing their perceived insider status, and consequently impacting their proactive actions (Ouyang et al., 2015). To establish causality and corroborate the findings from the field study, we carried out a scenario-based investigation.

### Study 2 method

#### Sample and procedure

Participants were recruited through the Wenjuanxing website (www.wjx.cn), which has been considered a reliable data source (Wu et al., 2020). 249 employees completed the experiment in exchange for a monetary payment (RMB ￥10; about U.S. $1.5) for their participation. Four participants did not pass the attention test and two did not report their gender. 44.4% of the participants were men. Most employees (82.7%) were aged from 26 to 40 years old, and 74.1% worked for less than 10 years.

We employed a 2 (high subordinate proactivity vs. low subordinate proactivity) × 2 (high subordinate in-role performance vs. low subordinate in-role performance) between-subjects experimental design. Specifically, participants were asked to imagine they were leaders of sales teams at ZK Inc. Their jobs were to supervise five employees’ daily work, monitor sales performance, and participate in the formulation and implementation of sales plans. The members were rewarded based on individual sales performance per month.

Employee proactive behavior was operationalized in the two scenarios. The scenarios were developed based on the items from the proactive behavior scale by Parker and Collins (2010). The behavioral content of employee proactivity included preventing possible problems, making suggestions, and taking charge.

In the high proactive behavior condition, participants read the following statement:

*In your view, Jamie is a person who often tries to adopt improved procedures for doing the job and brings about improved procedures for the work unit or department. Moreover, Jamie frequently tries to find the root cause of things that may go wrong and spends time on planning how to prevent reoccurring problems. Jamie also often speaks up with new ideas or changes in procedures and actively encourages others in the team to get involved with issues that affect the team*.

Participants in the low proactive behavior condition read the following:

*In your view, Jamie is a person who seldom tries to adopt improved procedures for doing the job and brings about improved procedures for the work unit or department. Moreover, Jamie scarcely tries to find the root cause of things that may go wrong and spends time planning how to prevent reoccurring problems. Jamie hardly ever speaks up with new ideas or changes in procedures and actively encourages others in the team to get involved with issues that affect the team*.

Next, we manipulated employee in-role performance conditions by describing the employee performance ranking in the team. Participants in the high in-role performance condition read that *in the recent sales figure of five subordinates, Jamie’s sales performance ranked second, and monthly sales volume was only 2% lower than the first*. Participants in the low-performance condition read that *Jamie’s sales performance ranked fourth*.

Participants were randomly assigned to one of the four scenarios (high proactivity, high performance; high proactivity, low performance; low proactivity, high performance; low proactivity, low performance). After reading the scenario, participants responded to measures of future orientation, abusive supervision, and manipulation check questions.

#### Measures

##### Leader future orientation

Participants were asked to evaluate their own future orientation levels using the same five items in Study 1. They rated their agreements with each item on the seven-point Likert scale (1 = *strongly disagree*, 7 = *strongly agree*). Cronbach’s alpha was 0.70.

##### Abusive supervision

Like previous studies (e.g., Camps et al., 2020; Walter et al., 2015), we asked participants to rate their likelihood engaging five behaviors from Study 1. A sample item is “I may ridicule Jamie.” A seven-point Likert scale (1 = *strongly disagree*, 7 = *strongly agree*) was also adopted. Cronbach’s alpha was 0.80.

##### Manipulation checks

To check the effectiveness of our manipulations, we asked two questions for proactive behavior and two questions for performance. Example questions are: “In the scenario that you read earlier, do you think Jamie Fiske is proactive?” (1 = *yes*, 2 = *no*), “To what extent is Jamie proactive?” (1 = *not proactive at all*, 7 = *very proactive*), “In the scenario that you read earlier, do you think Jamie’s performance is good?” (1 = *yes*, 2 = *no*), “How well does Jamie perform?” (1 = *very badly*, 7 = *very well*).

### Study 2 results

#### Manipulation checks

Participants reported the subordinate acted more proactively in the high-proactive behavior condition (M = 5.98, SD = 1.17) than in the low-proactive behavior condition (M = 3.14, SD = 1.45), *F*(1, 241) = 283.85, *p* < 0.001. Moreover, the subordinate was evaluated to perform better in the high-in-role performance condition (M = 5.44, SD = 1.16) than in the low in-role performance condition (M = 3.90, SD = 1.50), *F*(1, 241) = 80.29, *p* < 0.001. These results showed our manipulations were effective.

#### Tests of hypotheses

As illustrated in Table 5, the t-test results revealed that participants in the high proactive behavior condition (M = 2.32, SD = 0.90) reported lower levels of abusive supervision in comparison to those in the low proactive behavior condition (M = 3.28, SD = 1.02), t(241) = 7.79, *p* < 0.01, Cohen’s *d* = 0.88. Thus, H1 was supported.

**Table 5:** Independent samples test for abusive supervision (Study 2).

Condition	Mean	SD	*t*
High employee proactive behavior	2.32	0.90	7.79^**^
Low employee proactive behavior	3.28	1.02	

^**^p < 0.01.

A two-way analysis of variance (ANOVA) is used to examine the employee proactive behavior × manager future orientation interaction predicted in H2.

Participants’ future orientation scores were standardized prior to the analysis. As shown in Table 6, results indicated that the two-way interaction was not significantly related to abusive supervision (*B* = −0.08, ns). Hence, H2 was not supported.

**Table 6:** Analysis-of-variance results for abusive supervision (Study 2).

Predictor	Abusive supervision	
	*F*	*η* ^2^
Employee proactive behavior	59.70^**^	0.20
Supervisor future orientation	2.70	0.01
Employee proactive behavior × Supervisor future orientation	1.73	0.01

^**^p < 0.01.

We use general linear modeling analysis to examine the three-way interaction predicted in H3. As shown in Table 7, there were no significant two-way interactions. However, the three-way interaction of employee proactive behavior, employee performance, and supervisor future orientation was significant (*B* = −0.15, *p* < 0.05).

**Table 7:** General linear model results for abusive supervision (Study 2).

Predictor	Abusive supervision	
	*F*	*η* ^2^
Employee proactive behavior	64.86^**^	0.22
Supervisor future orientation	4.92^*^	0.02
Employee in-role performance	1.05	0.00
Proactive behavior × Future orientation	0.58	0.00
Proactive behavior × Employee in-role performance	0.41	0.00
Future orientation × Employee in-role performance	1.65	0.01
Proactive behavior × Future orientation × Employee in-role performance	5.20^*^	0.02

^**^p < 0.01, ^*^p < 0.05.

**Figure 3:**
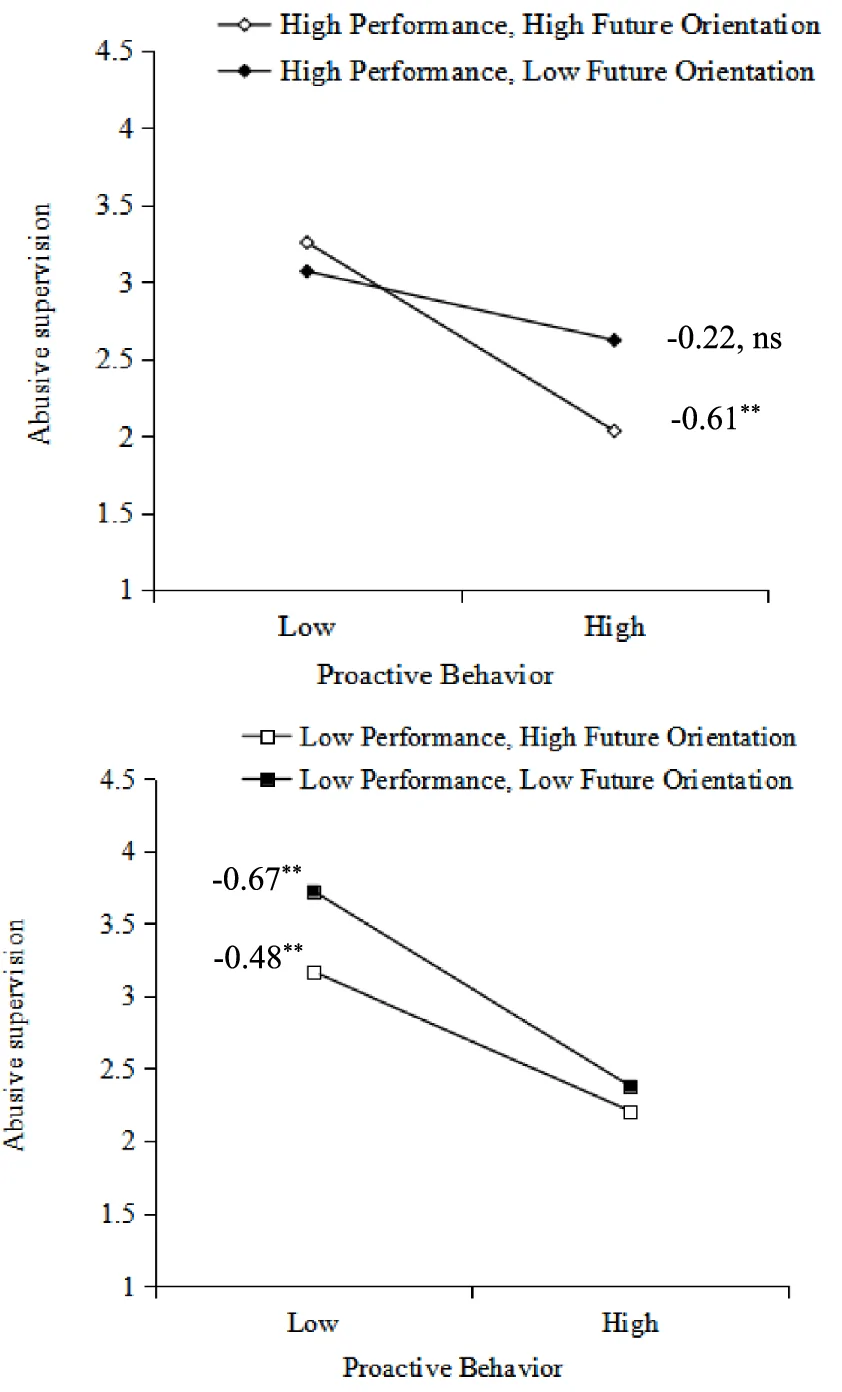
Three-way interaction effect on abusive supervision (Study 2).

The three-way interaction was plotted and illustrated in Figure 3. Results of simple slope analysis revealed that the relationship between employee proactive behavior and abusive supervision was significantly negative under the condition of high employee in-role performance and high supervisor future orientation (*β* = −0.61, *p* < 0.01), the condition of low employee in-role performance and high supervisor future orientation (*β* = −0.48, *p* < 0.01), and the condition of low employee in-role performance and low supervisor future orientation (*β* = −0.67, *p* < 0.01). However, the relationship was insignificant under the condition of high employee in-role performance and low supervisor future orientation (*β* = −0.22, ns).

Slope difference analysis indicated that there was a significant slope difference when employee in-role performance was high and manager future orientation was high versus when employee in-role performance was high and manager future orientation was low (Slopes 1 and 2; *t* = −2.19, *p* < 0.05). In contrast, the slope difference was not statistically significant for subordinates with low in-role performance (Slopes 3 and 4; *t* = 1.02, ns). Consequently, H3 was supported in Study 2. These findings were consistent with Study 1.

## General discussion

Consistent with our predictions, two studies indicate that: (1) leaders tend to exhibit less hostility towards employees who display more proactive behaviors; (2) this tendency is particularly pronounced among future-oriented leaders when employees’ in-role performance is high; (3) this tendency persists, albeit in a diminished form, for future-oriented leaders when employees’ in-role performance is low; (4) this tendency disappears for leaders with low future orientation when employees’ in-role performance is high.

Inconsistent with the prediction outlined in Hypothesis 2, neither study observed a two-way moderating effect of leader future orientation and subordinate proactive behavior on supervisory abuse. One possible explanation is the existence of other boundary conditions (e.g., task characteristics, environmental uncertainty, change-oriented organizational culture). This moderating effect becomes observable only when specific prerequisites are met (e.g., leaders facing high levels of task complexity and uncertainty). Another possible reason is the failure to account for control variables related to leadership (e.g., Machiavellianism, leader trait anger, Bhattacharjee and Sarkar, 2024), which may suppress the influence of leader future orientation, making it difficult to detect the moderating effect.

### Theoretical implications

Our results contribute to the extant literature on proactivity and abusive supervision in several ways. First, this study makes contributions to the proactive behavior literature by exploring the bottom-up effects of employees’ proactivity on their leaders’ behaviors, thereby addressing the call for more research into the consequences of employee proactive behavior (Parker et al., 2019; Pingel et al., 2019; Smith et al., 2026). Regarding the effect of proactive employee behavior on leadership responses and behaviors, existing studies primarily focus on performance evaluations and ratings of helping behaviors. A limited number of studies have investigated other consequential leader behaviors, such as voice endorsement, information provision, emotional support (Han et al., 2019; Shih and Nguyen, 2025; Xu et al., 2023). Highlighting the inhibitive impact of follower proactive behavior on supervisory abuse, the current study points out a new potential direction for employee proactive behavior in reducing social mistreatment from their supervisors.

Second, we extend the abusive supervision literature by further investigating what and how employees’ work behaviors may exert an influence on their supervisors’ acts of abuse or mistreatment (e.g., Lyubykh et al., 2022; Walter et al., 2015; Wee et al., 2017). Instead of concentrating on inducing factors (e.g., employee deviance, counterproductive behavior, and aggression), this research reveals that proactive actions taken by employees lead to greater recognition from their supervisors and result in fewer frequencies of abusive treatment towards them. In other words, proactive behavior serves as an active and effective strategy for employees to deal with abusive supervisors. This study enriches an emerging research stream that has begun to investigate which active behaviors exhibited by employees can mitigate abusive supervision from the perspective of followership and further broaden the nomological network of abusive supervision.

Third, our research enriches the boundary conditions under which employee positive behavior leads to favorable outcomes. Previous studies have shown that supervisors’ responses to subordinate proactivity can be influenced by factors such as supervisors’ regulatory focus, status goal striving, rule governance, and originality (Burris et al., 2022; Xu et al., 2023). By elucidating the moderating roles of supervisor future orientation and employee in-role performance, the present study addresses to the call made by Parker et al. (2019) and generates new insights into the social and relational boundary conditions for wise proactivity.

### Practical implications

This study offers three key managerial implications. First, employee proactivity not only promotes innovation and enhances performance at the work unit level, but also reduces managerial hostility, thereby preventing the emergence of unethical or hostile managerial environments. Hence, human resource management practices should prioritize the cultivation of employee proactivity. For instance, organizations could consider incorporating proactive personality assessments into the employee selection process to identify and hire individuals with a predisposition toward proactivity. Additionally, training programs designed to enhance employees’ self-efficacy (e.g., as proposed by Eden and Aviram, 1993) can be employed to increase proactive behaviors, particularly in fostering a ‘can do’ attitude (Parker et al., 2010). Organizations can enhance employees’ ‘reason to’ and ‘energized to’ motivations in exhibiting proactive behaviors through various means, including promotions, public recognition, monetary rewards, and the cultivation of a positive emotional climate. Such efforts not only improve organizational effectiveness but also reduce the likelihood of hostility and tension within manager-employee relationships.

Second, a future-oriented manager not only demonstrates more proactive goal setting and achievement but also shows a greater recognition of employee proactivity, particularly when employees display high in-role performance. Therefore, while organizations strive to enhance employee proactivity, it is equally important for them to concentrate on elevating their managers’ future orientation levels. Potential management practices to achieve this objective include selecting managers who possess a future-oriented mindset, cultivating a culture of future orientation and long-term thinking within the organization, integrating future orientation into managerial competencies, and incorporating long-term performance indicators into evaluations of managerial performance. Furthermore, it is important to note that employee proactivity could be perceived as threatening by some leaders, especially for those with low future orientation. Therefore, organizations need to consider such risks when encouraging employees to be proactive.

Finally, our findings, unfortunately, reveal that the average level of abusive supervision has not significantly decreased compared to studies conducted a decade ago (e.g., Walter et al., 2015). In some instances, it has even shown a slight increase (e.g., Tepper et al., 2011). Therefore, we continue to advocate for measures aimed at reducing hostile behaviors exhibited by supervisors toward employees. On the one hand, regarding supervisors, organizations can select managers who exhibit low levels of Machiavellianism and trait anger. Additionally, they can reduce managerial stress and self-depletion by implementing employee assistance programs, and providing training for supervisors in emotional management skills. On the other hand, with respect to subordinates, organizations can take several measures. They may filter out employees who possess dangerous worldviews or exhibit low capabilities. In addition, they can enhance employees’ ability to manage supervisor-subordinate relationships through interpersonal communication training. Furthermore, establishing grievance channels for employee relations is essential to protect employees.

### Limitations and future research

As with all studies, the present research has some limitations. First, this research employed the same measure of proactive work behavior integrated by Parker and Collins (2010) in our two studies, yielding similar results. However, other scholars in the field of proactivity have utilized different scales or objective measures in their studies (Pingel et al., 2019). Future research could retest our hypotheses by using a different proactive behavior scale.

The second limitation is that data were collected in China, which may restrict the generalizability of our findings to other cultural contexts. Chinese hold a relatively high level of power distance value, suggesting that the predicting power of subordinate proactivity on supervisory abuse may vary for individuals in other countries such as the USA, which is a low power distance society. We call for future research to replicate the present investigation across diverse cultural contexts.

Third, although Study 2 employs a vignette experiment to address causality, scenario-based designs may lack ecological validity and may not fully capture real-world managerial behavior. The artificial nature of the task in Study 2, where participants imagine themselves as leaders, could affect the external validity of the results. Therefore, we suggest that future study should invite real-world supervisors to participate in experiments. Moreover, ratings for employee proactivity and in-role performance in Study 1 were reported by supervisors at the same point of time, although this had little or no impact on the hypothesized three-way interaction (Podsakoff et al., 2012), we suggest that future research should consider using different scales or objective measures and collecting ratings at separate time points.

Fourth, our hypothesized two-way interaction between supervisor future orientation and subordinate proactive behavior was not supported in either study. This suggests that other unmeasured variables, such as task characteristics or supervisor traits like Machiavellianism or trait anger, may play a role. The absence of this effect points to the need for more nuanced models and additional control variables in future research. Furthermore, the potential for reverse causality and alternative explanations for the relationship between abusive supervision and employee proactivity is not fully excluded in two studies. Future studies should empirically disentangle the directionality of effects.

Finally, future research could also delve deeper into some intriguing yet somewhat counter-intuitive findings presented in the current study. In both studies, we found that even when employee performance was low, their proactivity still contributed to a reduction in abusive supervision, particularly for supervisors with a high future orientation. We encourage future research to replicate this finding using different samples and to investigate potential underlying mechanisms (e.g., positive expectation violation, Burgoon et al., 1995). Moreover, future research could further explore whether, how, why, and when employees’ proactive behaviors influence other forms of destructive leadership (e.g., exploitative leadership, Emmerling et al., 2023).

## Conclusion

In line with the followership tenet that leader behaviors are largely shaped by follower behaviors, the present study has found that supervisory abuse is reduced by follower proactive behaviors. This inhibiting effect of follower proactive behavior on supervisory abuse is contingent on the leader’s future orientation and follower in-role performance. In the case of high leader future orientation and high follower in-role performance, this effect is more significant. These findings extend our understanding of coping strategies for abusive supervision and managerial responses to employee proactive behavior.

## Data Availability

The raw data supporting the conclusions of this article will be made available by the authors, without undue reservation.
